# Comparison of External Oblique Intercostal Plane Block and Subcostal Transversus Abdominis Plane Block for Postoperative Analgesia After Open Radical Gastrectomy: A Single-Blind Randomized Trial

**DOI:** 10.3390/jcm15197736

**Published:** 2026-10-08

**Authors:** Uson Mamasaliev, Ahmet Taşar, Ersin Sönmez, Gamze Talih, Ayşe Ülgey

**Affiliations:** 1 Department of Anesthesiology and Reanimation, Liv Topkapı Hospital, İstinye University, İstanbul 34010, Turkey; uson.mamasaliev@gmail.com; 2 Anesthesiology and Reanimation Clinic, Sorgun State Hospital, Yozgat 66700, Turkey; ahmettasar@erciyes.edu.tr; 3 Department of Anesthesiology and Reanimation, School of Medicine, Erciyes University, Kayseri 38030, Turkey; ersinsonmez@erciyes.edu.tr (E.S.); aulgey@erciyes.edu.tr (A.Ü.)

**Keywords:** analgesia, postoperative pain, regional anesthesia, opioids, morphine

## Abstract

**Background:** Ultrasound-guided fascial plane blocks are increasingly incorporated into multimodal analgesia for upper abdominal surgery. Although both the external oblique intercostal plane (EOIP) block and the subcostal transversus abdominis plane (scTAP) block have been used for postoperative analgesia, evidence directly comparing their efficacy after open radical gastrectomy is lacking. We compared the postoperative analgesic efficacy of bilateral EOIP and bilateral scTAP blocks in patients undergoing open radical gastrectomy. **Methods:** In this prospective, single-center, randomized, patient-blinded trial, 60 adults undergoing elective open radical gastrectomy were randomly assigned to receive either a bilateral ultrasound-guided EOIP block or a bilateral scTAP block. The primary outcome was cumulative morphine consumption during the first 24 postoperative hours. Secondary outcomes included postoperative Numeric Rating Scale (NRS) scores, rescue analgesic requirements, postoperative nausea and vomiting (PONV), and length of hospital stay. **Results:** Thirty patients were analyzed in each group. 24 h cumulative morphine consumption did not differ significantly between the EOIP and scTAP groups (24.3 ± 11.4 mg vs. 22.4 ± 8.8 mg, respectively; mean difference 1.9 mg, 95% confidence interval −3.10 to 6.97; *p* = 0.47). Morphine consumption at 2, 6, and 12 h also showed no statistically significant differences. Postoperative NRS scores showed no significant differences at any time point. Rescue analgesic requirements and the proportion of patients experiencing PONV episodes did not differ significantly between the groups. No complications were observed. **Conclusions:** Among patients undergoing open radical gastrectomy, no statistically significant differences were detected between bilateral EOIP and bilateral scTAP blocks in postoperative opioid consumption, pain scores, rescue analgesic requirements, or PONV. These findings do not establish equivalence or efficacy relative to standard care without a block and warrant confirmation in larger multicenter trials.

## 1. Introduction

Radical gastrectomy continues to be the main curative option for patients with resectable gastric cancer [1]. Open surgery and extensive visceral manipulation may lead to severe postoperative pain [2]. Inadequate control of this pain delays mobilization, increases PONV, prolongs hospital stay, and contributes to the development of chronic pain [3]. Therefore, postoperative pain management is crucial to improving patient comfort and facilitating recovery.

Opioids have long been the primary option for managing pain. However, dose-dependent adverse effects such as respiratory depression, urinary retention, pruritus, PONV, and sedation limit their use as sole analgesic agents [4]. Therefore, opioid-sparing multimodal analgesia incorporating regional anesthesia techniques has become an integral component of perioperative pain management [5]. Thoracic epidural analgesia has long been considered the gold standard; however, it is associated with hypotension and potential neurological complications [6].

Ultrasound-guided fascial plane blocks have emerged as attractive alternatives to neuraxial techniques for postoperative analgesia after upper abdominal surgery because of their favorable safety profile and relative technical simplicity [7,8]. The scTAP block was developed to provide analgesia to the supraumbilical anterior abdominal wall and has been used in a variety of upper abdominal procedures, including gastric surgery [9,10]. More recently, the EOIP block has been introduced as a novel interfascial plane block targeting the lower thoracoabdominal nerves. Anatomical studies have demonstrated cranio-caudal spread along the external oblique–intercostal fascial plane with involvement of both the anterior and lateral cutaneous branches of the thoracoabdominal nerves [11,12]. Clinical studies have subsequently suggested a potential analgesic role for the EOIP block in upper abdominal surgery [13,14]. However, despite increasing clinical interest in both techniques, direct comparative evidence remains limited, particularly in patients undergoing standardized open upper abdominal surgery.

In this study, we aimed to compare the analgesic efficacy of bilateral scTAP and bilateral EOIP block in postoperative pain after radical gastrectomy. Our primary aim was to compare cumulative opioid consumption at the postoperative 24th hour. Secondary aims were postoperative NRS pain scores, rescue analgesic use, and PONV.

## 2. Materials and Methods

This single-center, prospective, randomized, and single (patient) blind study received approval from the Erciyes University Clinical Research Ethics Committee (No. 2023/600—25 September 2023) and was registered on ClinicalTrials.gov (No. NCT06412796; https://www.clinicaltrials.gov) on 9 May 2024. Informed written consent was obtained from all participants prior to participation. All procedures took place at Erciyes University Hospital following the principles of the 2013 Declaration of Helsinki and the 2025 CONSORT guidelines.

The study included patients aged 18 or older with ASA physical status I–III who were scheduled for open radical gastrectomy between July 2024 and March 2025. All underwent open total gastrectomy with D2 lymph node dissection via midline laparotomy. The first patient was enrolled on 1 July 2024.

Patients were excluded from the study if they had any of the following conditions: (1) allergy to amide local anesthetics, (2) prior opioid use, (3) opioid dependence or addiction, (4) neuropathic pain and the use of medications such as gabapentinoids, tricyclic antidepressants, or serotonin-noradrenaline reuptake inhibitors, (5) alcohol dependence or addiction, (6) cognitive impairment, (7) psychiatric disorders, (8) previous abdominal surgery or procedures that could affect the injection’s spread to the block site, and (9) diabetes mellitus, due to the risk of diabetic peripheral neuropathy and altered pain perception.

### 2.1. Randomization and Blinding

Patients were randomly assigned to two groups based on a computer-generated list of random numbers (https://www.randomizer.org). The researcher (U.M.) prepared sealed, opaque envelopes for each patient based on this list. On the day of the procedure, the researcher (A.Ü.) opened the envelopes and allocated patients to either the scTAP or EOIP groups. Patients were unaware of their group allocation. Due to the nature of the interventions, the anesthesiologist (G.T.) performing the block could not be blinded. Postoperative pain assessments and data collection were carried out by investigators (A.T. and E.S.) who were not blinded to group assignments.

### 2.2. Subcostal Transversus Abdominis Plane (scTAP) Block

All block procedures were performed subsequent to induction and endotracheal intubation, prior to the incision. With the patient in the supine position and following sterile preparation, a 15 MHz linear ultrasound probe was positioned transversely just caudal to the xiphoid process to visualize the bilateral rectus muscles and linea alba. The probe was then advanced caudally and laterally, aligning with the costal margin at a slight oblique angle. Upon visualizing the linea semilunaris of the rectus muscle, the external oblique, internal oblique, and transversus abdominis muscles, an 80 mm block needle was introduced from superomedial to inferolateral using an in-plane technique. Confirmation of the fascia’s position between the rectus abdominis and transversus abdominis muscles was achieved via hydrodissection with 2 mL of saline, followed by the injection of 20 mL of 0.25% bupivacaine. This procedure was conducted identically on the contralateral side.

### 2.3. External Oblique Intercostal Plane (EOIP) Block

With the patient in the supine position, following sterile preparation, a 15 MHz linear ultrasound transducer was positioned obliquely medial to the anterior axillary line. The sixth and seventh ribs, along with the skin, subcutaneous tissues, and external oblique muscle, were identified. The needle was guided in-plane beneath the external oblique muscle. After administering 2 mL of saline to confirm correct needle placement, 20 mL of 0.25% bupivacaine was injected between the external oblique muscle and the intercostal muscles. This procedure was conducted identically on the contralateral side.

### 2.4. Anesthesia Management

In the operating room, patients were monitored in accordance with ASA guidelines utilizing electrocardiography, pulse oximetry, and non-invasive blood pressure measurements. Balanced crystalloids were started at a maintenance dose of 5–10 mL/kg. Pre-oxygenation was conducted two minutes prior to the induction of anesthesia using a face mask delivering oxygen at a flow rate of 6 L/min. Anesthesia induction was achieved through the intravenous administration of 1–2 mg/kg of propofol, 1–2 µg/kg of fentanyl, and 0.4–0.6 mg/kg of rocuronium bromide. Anesthesia was maintained with sevoflurane in a 50% oxygen-50% air mixture at 1 minimum alveolar concentration, combined with remifentanil at a rate of 0.05–0.5 µg/kg/min. Additional muscle relaxation was administered as required via intravenous bolus of 0.1 mg/kg of rocuronium bromide. At the end of anesthesia, 200 mg of sugammadex was administered intravenously to antagonize muscle relaxation, and patients were extubated once meeting the extubation criteria. Standard monitoring continued in the post-anesthesia care unit (PACU), during which patients were re-informed regarding the use of the patient-controlled analgesia (PCA) device. Patients were subsequently transferred to the general surgery ward after a period of 60–90 min in the postoperative care unit, upon achieving modified Aldrete scores of 9 or higher.

### 2.5. Postoperative Analgesia and PONV Management

Throughout the study, all patients received a standardized protocol for postoperative pain management. Each patient was administered 1 g of paracetamol and 0.05 mg/kg of morphine intravenously approximately 20 min prior to the end of surgery. The morphine bolus was delivered via a PCA device and was incorporated into the cumulative morphine consumption. Paracetamol administration was continued in the ward at a dosage of 1 g every 8 h. To prevent postoperative nausea and vomiting, 1 mg of granisetron was administered 20 min before the end of the procedure.

The PCA device was configured to administer a 1 mg bolus dose of morphine on demand, with a lock-in interval of 15 min, and without basal infusion. The device was maintained in use for 24 h. In cases where patients experienced moderate to severe pain with an NRS score of 4 or higher, 50 mg of dexketoprofen was given intravenously as rescue analgesia. The maximum daily dose of dexketoprofen was 150 mg. All patients experiencing nausea lasting longer than 10 min postoperatively or any episodes of vomiting within the first 24 h after surgery received rescue antiemetic therapy with 1 mg of granisetron administered intravenously.

### 2.6. Outcome Measures

The primary outcome was cumulative morphine consumed within the first 24 h after surgery. Morphine consumption from the PCA device was recorded at 2, 6, 12, and 24 h postoperatively. Secondary outcomes encompassed postoperative pain scores, the requirement for rescue analgesics, the incidence of PONV, intraoperative remifentanil consumption (mg), and duration of hospital stay (days). Postoperative pain levels at rest were evaluated verbally using the NRS at 2, 6, 12, and 24 h after surgery. The NRS is an 11-point scale from 0, indicating no pain, to 10, representing the worst pain imaginable. Data on rescue analgesia from patient records were recorded at the same postoperative intervals. The number of patients requiring rescue analgesia was reported. PONV was primarily analyzed as a binary variable at the individual patient level (presence or absence within 24 h). Transient nausea lasting under 10 min that resolved on its own without intervention was not considered clinically significant PONV and was not recorded as an event.

### 2.7. Sample Size

The sample size was calculated using G*Power (version 3.1.9.6; Heinrich-Heine-Universität Düsseldorf, Düsseldorf, Germany) based on the primary outcome of cumulative 24 h postoperative morphine consumption. A previous study in patients undergoing radical gastrectomy reported a mean 24 h morphine consumption of 33 ± 11 mg following scTAP block, and this standard deviation was used for the sample size estimation [15]. A between-group difference of 10 mg in cumulative 24 h morphine consumption was prespecified as clinically relevant. This threshold was based on previous evidence from a systematic review and network meta-analysis of regional analgesic techniques in patients undergoing midline laparotomy, in which 10 mg of intravenous morphine equivalent consumption was used as the minimal clinically important difference (MCID) [16]. The review acknowledged that this threshold was empirical. However, because a validated procedure-specific MCID for postoperative morphine consumption after radical gastrectomy has not been established, the 10 mg threshold was regarded as a prespecified clinically relevant difference rather than a validated gastrectomy-specific MCID. With an assumed standard deviation of 11 mg, this corresponded to a Cohen’s d of 0.91. Assuming a two-sided α of 0.05 and 90% power, 27 patients were required in each group. To account for an anticipated 10% dropout rate, 30 patients were enrolled in each group.

### 2.8. Statistical Analysis

Data were analyzed and visualized using RStudio (version 2024.12.1; Posit Software, PBC, Boston, MA, USA). Normality was assessed using histograms and the Shapiro–Wilk test. Continuous variables were presented as mean ± standard deviation (SD) or median (interquartile range [IQR]), as appropriate, whereas categorical variables were summarized as counts and percentages. Normally distributed continuous variables were compared using the independent-samples *t*-test, whereas non-normally distributed variables were compared using the Wilcoxon–Mann–Whitney test. Categorical variables were analyzed using Pearson’s chi-square test or Fisher’s exact test, as appropriate. For cumulative postoperative morphine consumption, between-group differences were expressed as mean differences with 95% confidence intervals (CIs) estimated using bias-corrected and accelerated bootstrap resampling (10,000) replicates. For postoperative pain scores, between-group differences were expressed as Hodges–Lehmann median differences with 95% CIs. All statistical tests were two-sided, and a *p*-value < 0.05 was considered statistically significant. Between-group differences are reported as EOIP minus scTAP.

## 3. Results

**Figure 1:**
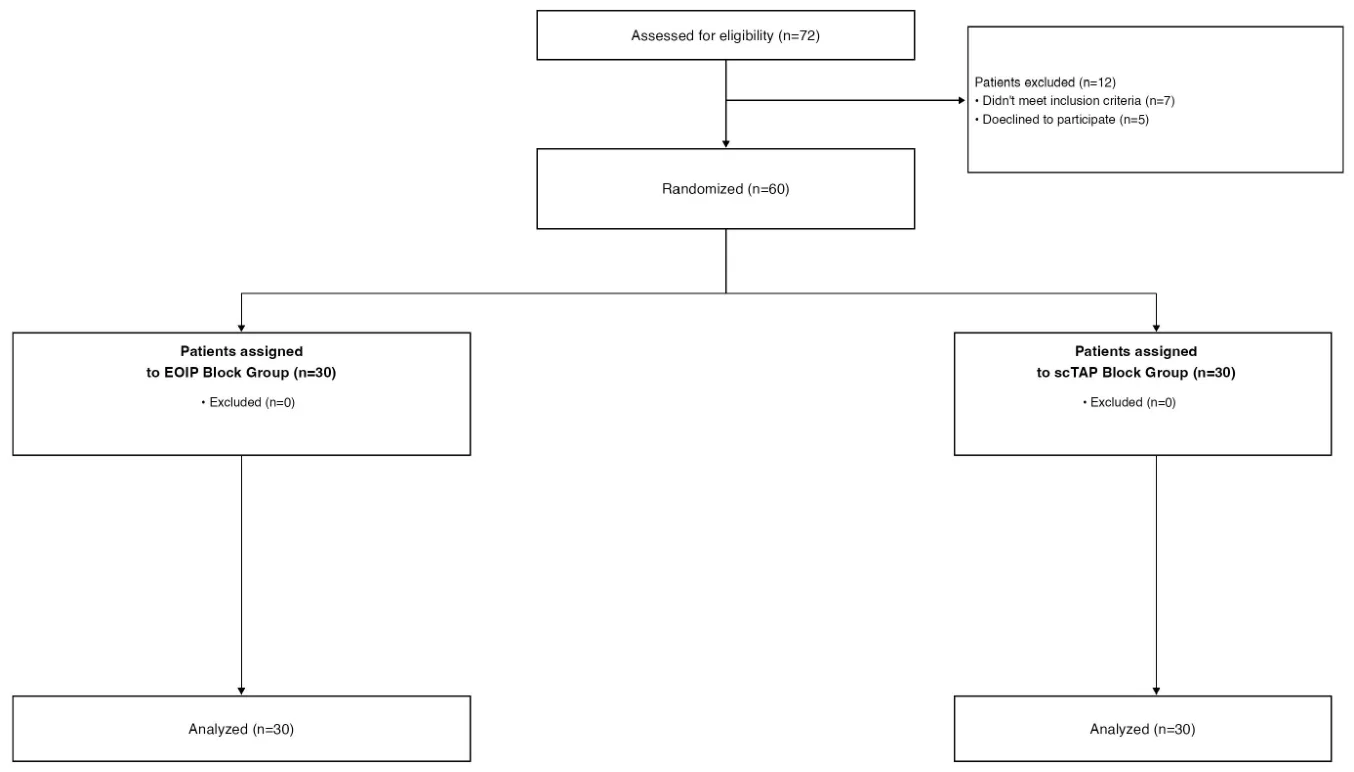
CONSORT flow diagram.

A total of 72 patients were assessed for eligibility, of whom 12 were excluded (7 did not meet the inclusion criteria and 5 declined to participate). Sixty patients were randomized, with 30 allocated to the bilateral EOIP block group and 30 to the bilateral scTAP block group. All randomized patients received the allocated intervention and completed follow-up; therefore, all 60 patients were included in the final analysis (Figure 1).

Baseline demographic and perioperative characteristics are presented in Table 1. No statistically significant between-group differences were detected in age, sex distribution, body mass index, duration of surgery, intraoperative remifentanil use, or length of hospital stay.

**Figure 2:**
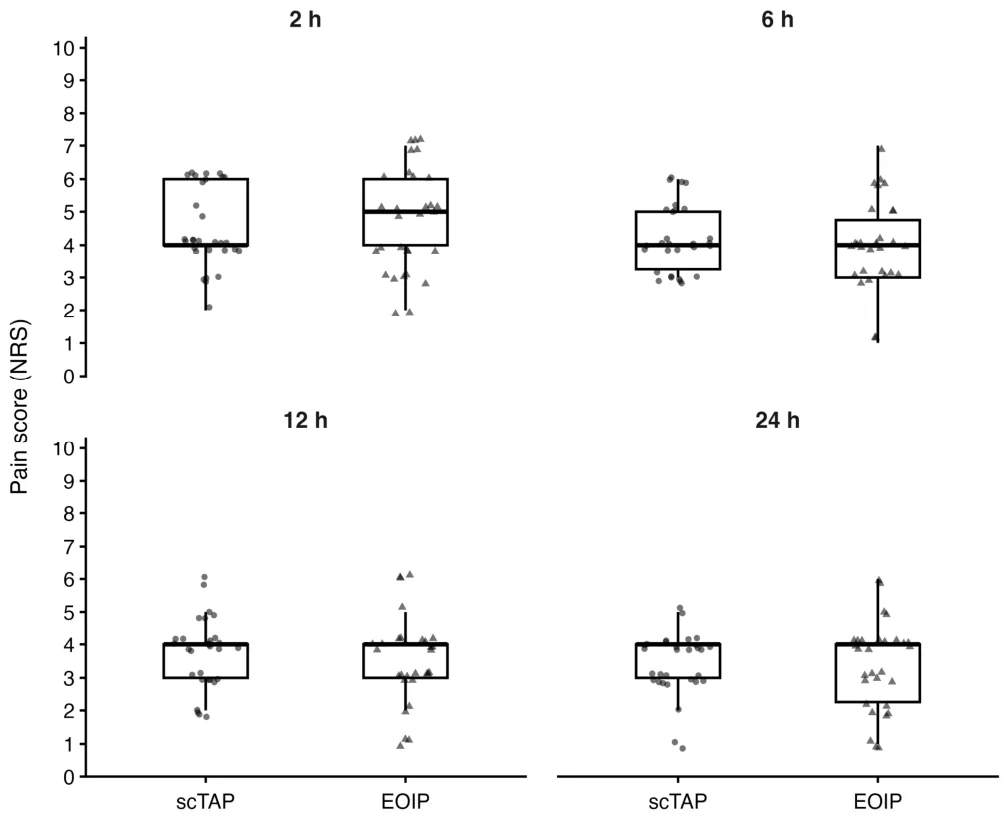
Distribution of postoperative Numeric Rating Scale (NRS) pain scores at 2, 6, 12, and 24 h following the scTAP or EOIP block. Box plots represent the median, interquartile range, and whiskers extending to 1.5× the interquartile range. Individual symbols represent patient-level observations distributed according to local data density. Circles denote the scTAP group and triangles denote the EOIP group.

Cumulative morphine consumption at the postoperative 24th hour, the primary outcome, did not differ significantly between the EOIP and scTAP groups (24.3 ± 11.4 mg vs. 22.4 ± 8.8 mg, respectively; mean difference (EOIP minus scTAP), 1.9 mg, 95% CI −3.10 to 6.97; *p* = 0.47). Similarly, cumulative morphine consumption at 2, 6, and 12 h also showed no statistically significant differences (Table 2). Postoperative NRS pain scores at 2, 6, 12, and 24 h did not differ significantly between the groups. Hodges–Lehmann median differences were close to zero at all postoperative time points, and none of the comparisons reached statistical significance (Table 2, Figure 2).

No statistically significant between-group differences were detected in rescue analgesic requirements or the proportion of patients experiencing PONV within each postoperative interval (Table 3). No complications were observed in either group.

## 4. Discussion

The present randomized controlled trial found no statistically significant differences between the EOIP and scTAP groups in cumulative morphine consumption, postoperative pain scores, rescue analgesic requirements, PONV incidence, antiemetic use, or length of hospital stay. Because the study did not include a no-block or standard-care control group, these findings should be interpreted as a direct comparison between the two techniques rather than evidence of the absolute analgesic efficacy of either block.

Pain after radical gastrectomy results from somatic and visceral nociceptive mechanisms. Somatic pain primarily originates from the upper midline laparotomy and abdominal wall retraction, whereas visceral pain is generated by gastric resection, extensive lymphadenectomy, and manipulation of intra-abdominal organs. Thoracic epidural analgesia (TEA) has long been considered the reference regional analgesic technique for major upper abdominal surgery because it provides effective analgesia by targeting somatic and visceral pain generators [15,17]. However, TEA requires technical expertise and is associated with adverse events, including hypotension, urinary retention, motor blockade, epidural hematoma, and limitations in patients receiving perioperative anticoagulant therapy [18,19,20]. These drawbacks have spurred growing interest in ultrasound-guided fascial plane blocks as simpler, potentially safer alternatives within multimodal analgesic protocols.

TAP blocks have become an integral part of multimodal analgesia after abdominal surgery. The scTAP block is a modification designed to block the supra-umbilical anterior abdominal wall. Most available evidence on the scTAP block comes from laparoscopic upper abdominal procedures, whereas studies evaluating its use in open abdominal surgery are relatively limited. Nevertheless, the available evidence suggests that scTAP provides superior postoperative analgesia and opioid-sparing effects compared with intravenous analgesia alone [21,22,23,24].

The EOIP block has recently emerged as an alternative approach for upper abdominal surgery. Initial findings on cadavers demonstrated cranio-caudal spread of the injectate across the T7–T10 intercostal levels, with staining of both the anterior and lateral cutaneous branches of the intercostal nerves [12]. Subsequent clinical studies have reported favorable postoperative analgesic outcomes following the EOIP block in various upper abdominal procedures, including laparoscopic cholecystectomy, hepatobiliary surgery, and upper gastrointestinal surgery [13,25].

To date, only a limited number of studies have directly compared the EOIP block with other upper abdominal fascial plane blocks. In a randomized trial comparing unilateral EOIP and scTAP blocks in patients undergoing supraumbilical surgery through a subcostal incision, the primary outcome, time to first analgesic request, was significantly longer in the EOIP block group. In addition, although the EOIP block was associated with lower postoperative opioid consumption and higher patient satisfaction, these were secondary outcomes for which the study was not specifically powered. Therefore, these findings should be interpreted cautiously [26]. Another randomized trial involving open upper abdominal surgery found that both the EOIP and scTAP blocks were associated with lower intraoperative and postoperative opioid consumption than the control group. However, although the EOIP block was associated with lower intraoperative fentanyl requirements, postoperative opioid consumption did not differ between the two block groups [27]. The discrepancy between these studies may be partly explained by differences in surgical characteristics, particularly incision type. All patients in the first study underwent surgery via a unilateral subcostal incision, whereas the latter study included patients undergoing midline and lateral upper abdominal incisions. Because the EOIP block has been shown to provide more extensive anterolateral abdominal wall coverage through blockade of the lateral cutaneous branches of the thoracoabdominal nerves, its potential analgesic advantage may be more pronounced for unilateral subcostal incisions than for more heterogeneous incision patterns. This hypothesis is further supported by clinical studies evaluating postoperative cutaneous sensory distribution in patients undergoing laparoscopic cholecystectomy, which demonstrated that the sensory loss produced by the scTAP block was largely confined to the periumbilical region, whereas the EOIP block extended laterally over the anterolateral abdominal wall [28,29].

To our knowledge, this is the first randomized trial directly comparing bilateral EOIP and bilateral scTAP blocks exclusively in patients undergoing open radical gastrectomy through a standardized midline laparotomy. By evaluating a homogeneous surgical population, our study minimized variability arising from differences in surgical procedures and incision characteristics. Within this standardized surgical setting, postoperative opioid consumption and all other clinically relevant analgesic outcomes were not statistically different between the EOIP and scTAP groups. Similarly, a randomized trial comparing bilateral EOIP and bilateral scTAP blocks in living donor hepatectomy, another homogeneous surgical population, also demonstrated no significant differences in postoperative opioid consumption, pain scores, rescue analgesic requirements, or PONV [30]. Overall, these results suggest that the effectiveness of EOIP and scTAP blocks for pain relief may depend on surgical details such as incision type, rather than indicating that one method is consistently better than the other.

This study had several limitations. First, the lack of a no-block or standard-care control group precludes conclusions regarding the absolute analgesic efficacy of either technique. Therefore, the present findings only allow comparison of postoperative outcomes between the EOIP and scTAP groups and should not be interpreted as evidence that either block is effective compared with standard care. Second, since the blocks were carried out after general anesthesia induction, dermatomal sensory assessment could not be performed to verify the effectiveness of the blockade. Consequently, unrecognized block failures cannot be excluded and may have attenuated the observed differences between the groups. In addition, procedural characteristics such as block performance time, number of needle passes, and technical difficulty were not prospectively recorded. Therefore, the technical performance of the two block techniques could not be formally compared. Finally, the single-center design and exclusion of diabetic patients may limit the generalizability of our findings to other institutions and patient populations.

## 5. Conclusions

In this relatively small, single-center randomized trial of patients undergoing open radical gastrectomy, no statistically significant differences were detected between bilateral EOIP and bilateral scTAP blocks in postoperative opioid consumption, pain scores, rescue analgesic requirements, PONV, or length of hospital stay. These findings warrant confirmation in larger multicenter studies, while future trials including a no-block or standard-care control group are needed to establish the absolute analgesic efficacy of these techniques.

## Figures and Tables

**Table 1:** Demographics and perioperative characteristics.

	External Oblique Intercostal Plane Block	Subcostal Transversus Abdominis Plane Block	*p* Value
Sample size, *n*	30	30	
Mean age (SD) in years	64.8 (11.9)	61.4 (9.8)	0.23
Mean body mass index (SD), (kg/m^2^)	26.9 (4.5)	27.3 (4.4)	0.75
Sex, n (%)			
Male	23 (77)	23 (77)	>0.99
Female	7 (23)	7 (23)	
Mean surgical duration (SD), min	162.2 (49.9)	165.3 (39.2)	0.79
Median intraoperative remifentanil consumption (IQR), (mg)	0.4 (0.3–0.6)	0.5 (0.3–0.6)	0.50
Median length of hospital stay (IQR) in days	7.0 (5.0–10.0)	7.0 (6.0–8.8)	0.93

**Table 2:** Postoperative opioid consumption and pain scores.

	External Oblique Intercostal Plane Block	Subcostal Transversus Abdominis Plane Block	Between-Group Difference (95% CI)	*p*-Value
Sample size, n	30	30		
Mean cumulative postoperative morphine (SD), mg				
2nd hour	4.5 (2.2)	5.0 (2.4)	−0.47 (−1.67 to 0.63)	0.43
6th hour	10.3 (4.6)	10.3 (4.5)	−0.07 (−2.27 to 2.26)	0.96
12th hour	17.0 (7.3)	16.2 (6.5)	0.83 (−2.57 to 4.30)	0.65
24th hour	24.3 (11.4)	22.4 (8.8)	1.9 (−3.10 to 6.97)	0.47
Median postoperative pain score (IQR), NRS 0–10				
2nd hour	5.0 (4.0–6.0)	4.0 (4.0–6.0)	0 (−1.00 to 1.00)	0.45
6th hour	4.0 (3.0–4.8)	4.0 (3.2–5.0)	0 (−1.00 to 0.00)	0.62
12th hour	4.0 (3.0–4.0)	4.0 (3.0–4.0)	0(−1.00 to 0.00)	0.39
24th hour	4.0 (2.2–4.0)	4.0 (3.0–4.0)	0 (−1.00 to 0.00)	0.83

CI: Confidence interval; NRS, Numeric rating scale.

**Table 3:** Secondary outcomes.

	External Oblique Intercostal Plane Block	Subcostal Transversus Abdominis Plane Block	*p* Value
Sample size, n	30	30	
Rescue analgesic requirement, n (%)			
0–2nd hour	8 (27)	6 (20)	0.54
2–6th hour	4 (13)	8 (27)	0.20
6–12th hour	3 (10)	6 (20)	0.28
12–24th hour	5 (17)	3 (10)	0.71
Patients experiencing at least one episode of PONV, n (%)			
0–2nd hour	1 (3)	2 (7)	0.50
2–6th hour	1 (3)	5 (17)	0.09
6–12th hour	4 (13)	1 (3)	0.35
12–24th hour	0 (0)	1 (3)	0.50

PONV, Postoperative nausea and vomiting.

## Data Availability

The data presented in this study are available from the corresponding author upon reasonable request. The data are not publicly available due to privacy and ethical restrictions related to participant-level clinical data.
